# Supplementary material for: Quantitative single-cell analysis of Leishmania major amastigote differentiation demonstrates variably extended expression of the lipophosphoglycan (LPG) virulence factor in different host cell types
Source: PLoS Negl Trop Dis. 2022 Oct 27;16(10):e0010893. doi: 10.1371/journal.pntd.0010893 (PMC9642900; doi:10.1371/journal.pntd.0010893)
Supplement: S1 Fig — (A) Flow cytometric analysis of YFP signal from WT L. major and L. major expressing YFP from the small ribosomal subunit locus under log-phase (procyclic promastigotes) or stationary-phase (includes metacyclic promastigotes) conditions. (B) Western blot analysis of YFP expression in log phase and stationary phase parasites stably transfected with YFP transgene. L. major histone H2A levels were assessed as a protein loading control. (C) The abundance of YFP mRNA in log and stationary phase parasites was determined by q-RT-PCR. *, P < 0.05. Abbreviations: p, parasite posterior; n, nucleus; k, kinetoplast. (PDF) [file pntd.0010893.s001.pdf]

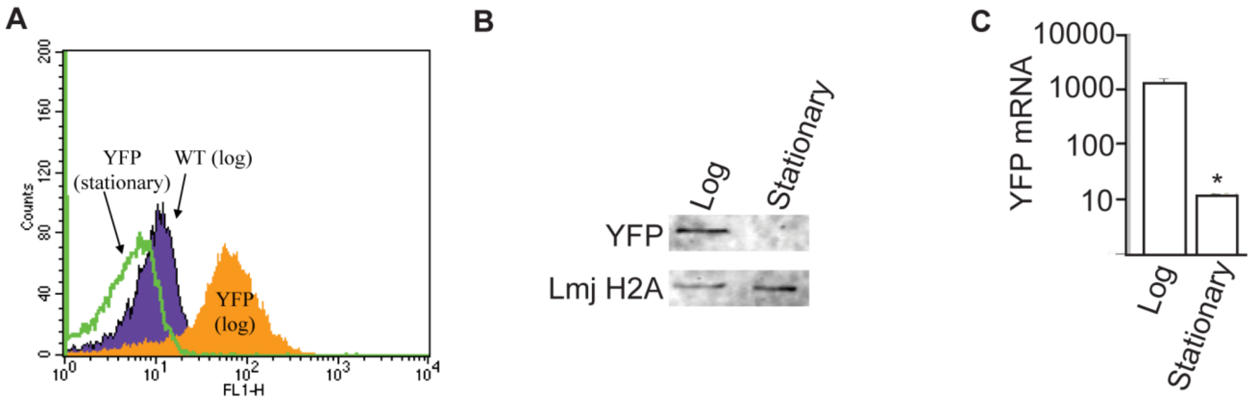

S1 Fig. Developmental regulation of YFP reporter.

(A) Flow cytometric analysis of YFP signal from WT *L. major* and *L. major* expressing YFP from the small ribosomal subunit locus under log-phase (procyclic promastigotes) or stationary-phase (includes metacyclic promastigotes) conditions. **(B)** Western blot analysis of YFP expression in log phase and stationary phase parasites stably transfected with YFP transgene. *L. major* histone H2A levels were assessed as a protein loading control. **(C)** The abundance of YFP mRNA in log and stationary phase parasites was determined by q-RT-PCR. *, $P < 0.05$. Abbreviations: p, parasite posterior; n, nucleus; k, kinetoplast.
