# Supplementary material for: Quantitative single-cell analysis of Leishmania major amastigote differentiation demonstrates variably extended expression of the lipophosphoglycan (LPG) virulence factor in different host cell types
Source: PLoS Negl Trop Dis. 2022 Oct 27;16(10):e0010893. doi: 10.1371/journal.pntd.0010893 (PMC9642900; doi:10.1371/journal.pntd.0010893)
Supplement: S2 Fig — (A) C57B6 mice were injected subcutaneously in footpad tissue with metacyclic stage parasites stably transfected with YFP transgene. 16 d later, mice were sacrificed and infected footpad tissue sectioned and stained to detect parasite histones (blue) and YFP. YFP-negative parasites are indicated by arrows. Scale bars, 5 μm. (B, C) PEMs were fixed 10 h after infection and stained with T17 (B) or T18 (C) antibodies (red). Parasites were identified based on nuclear staining with antibodies against parasite histones (blue). The percent of parasites positive for T17 or T18 reactivity and YFP fluorescence (green) was determined by analysis of confocal micrographs. Arrowheads indicate T17/T18 positive, YFP-negative parasites, and arrows indicate double-negative parasites. Scale bars, 5 μm. (PDF) [file pntd.0010893.s002.pdf]

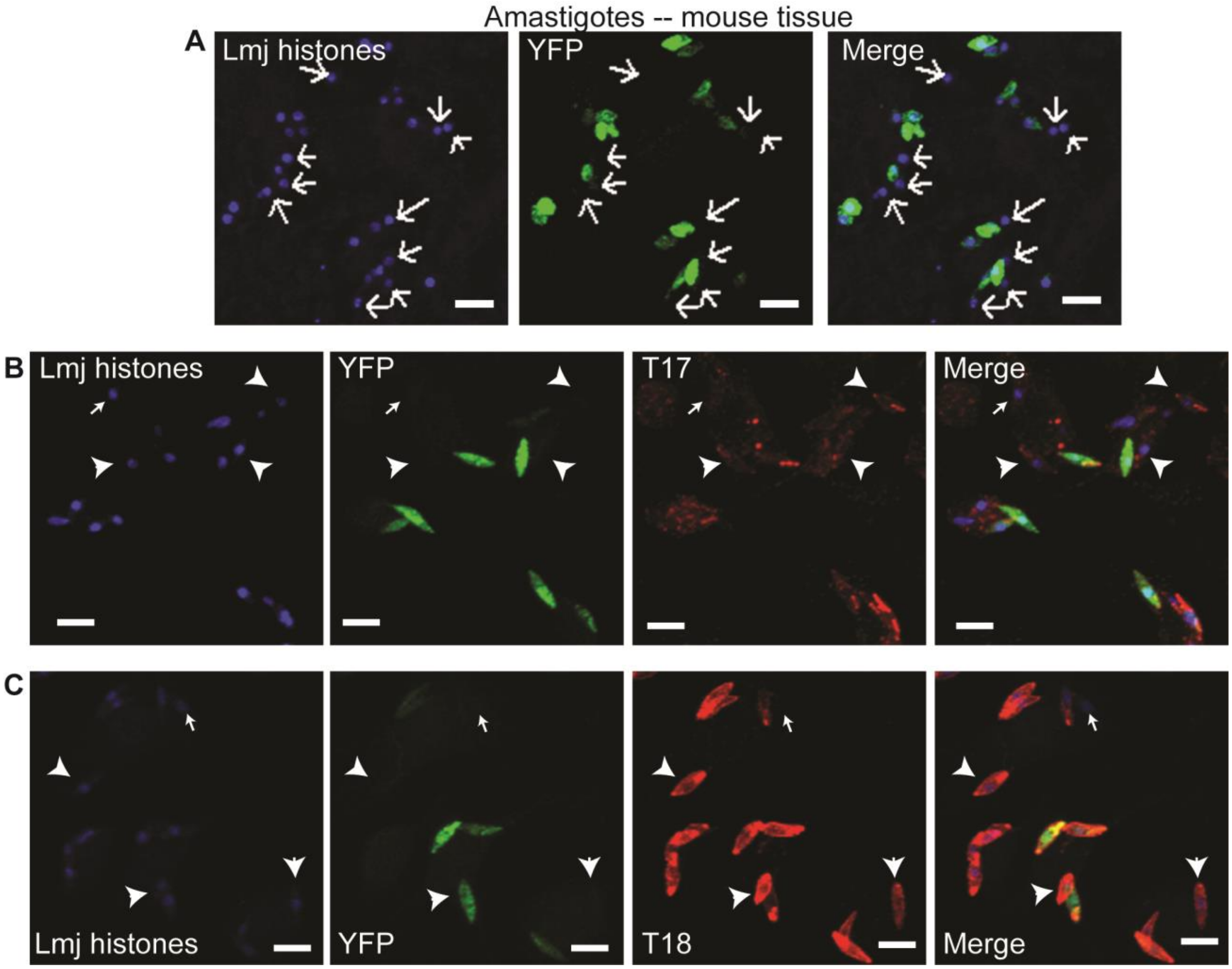

S2 Fig. mAb T17 and T18 reactivity precedes YFP expression during metacyclic-to-amastigote transition.

(A) C57B6 mice were injected subcutaneously in footpad tissue with metacyclic stage parasites stably transfected with YFP transgene. 16 d later, mice were sacrificed and infected footpad tissue sectioned and stained to detect parasite histones (blue) and YFP. YFP-negative parasites are indicated by arrows. Scale bars, 5 µm. **(B, C)** PEMs were fixed 10 h after infection and stained with T17 (B) or T18 (C) antibodies (red). Parasites were identified based on nuclear staining with antibodies against parasite histones (blue). The percent of parasites positive for T17 or T18 reactivity and YFP fluorescence (green) was determined by analysis of confocal micrographs. Arrowheads indicate T17/T18 positive, YFP-negative parasites, and arrows indicate double-negative parasites. Scale bars, 5 µm.
