# Supplementary material for: Quantitative single-cell analysis of Leishmania major amastigote differentiation demonstrates variably extended expression of the lipophosphoglycan (LPG) virulence factor in different host cell types
Source: PLoS Negl Trop Dis. 2022 Oct 27;16(10):e0010893. doi: 10.1371/journal.pntd.0010893 (PMC9642900; doi:10.1371/journal.pntd.0010893)
Supplement: S3 Fig — L. major promastigotes were cultured until the third day of stationary phase at 26°C, then placed in fresh media and cultured at 37°C for 24 hours prior to harvest and analysis. (A) Parasites were fixed and stained with the indicated markers prior to confocal microscopy. Micrographs were visually scored for PFR negativity or T17/T18 positivity. N > 200 parasites. (B) Flow cytometric analysis of YFP fluorescence of the starting culture of stationary phase parasites (green, 26°C) and parasites following 24 hours of culture at 37°C (purple). (C) Representative electron micrograph of a parasite following culture at 37°C that has typical amastigote morphology including a round shape and a spacious flagellar pocket. (PDF) [file pntd.0010893.s003.pdf]

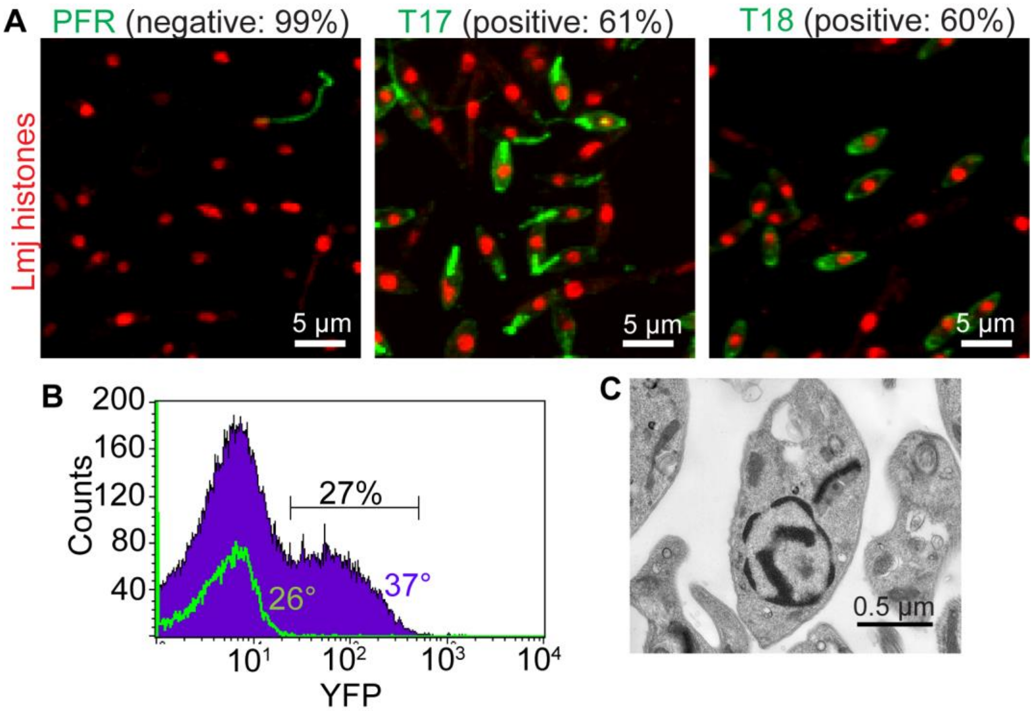

S3 Fig. Acquisition of amastigote traits by *L. major* cultured at 37º C in the absence of host cells.

L. major promastigotes were cultured until the third day of stationary phase at 26º C, then placed in fresh media and cultured at 37º C for 24 hours prior to harvest and analysis. **(A)** Parasites were fixed and stained with the indicated markers prior to confocal microscopy. Micrographs were visually scored for PFR negativity or T17/T18 positivity. N > 200 parasites. **(B)** Flow cytometric analysis of YFP fluorescence of the starting culture of stationary phase parasites (green, 26º C) and parasites following 24 hours of culture at 37º C (purple). **(C)** Representative electron micrograph of a parasite following culture at 37º C that has typical amastigote morphology including a round shape and a spacious flagellar pocket.
