# Supplementary material for: Quantitative single-cell analysis of Leishmania major amastigote differentiation demonstrates variably extended expression of the lipophosphoglycan (LPG) virulence factor in different host cell types
Source: PLoS Negl Trop Dis. 2022 Oct 27;16(10):e0010893. doi: 10.1371/journal.pntd.0010893 (PMC9642900; doi:10.1371/journal.pntd.0010893)
Supplement: S4 Fig — (A) PEMs were infected with L. major and harvested 2, 24, and 72 hours after infection. Ara-capped LPG is detected with mAB 3F12 (green), and parasite histones are shown in red. Data indicate the percent of parasites positive for 3F12 labeling out of the total number of parasites analyzed (N). Scale bar, 5 μm. (B) Representative image of parasites within BMM fixed 72 h post infection showing LPG and/or YFP positivity. Parasite nuclei are shown in blue. Arrows indicate LPG and YFP double-negative parasites. Scale bar, 5 μm. Analysis of images like that in (B) was performed to determine the LPG-positivity (C) or YFP-positivity (D) of total parasites or within the indicated parasite sup-populations. N = 362 parasites. N.S., not specific (Chi-square). (PDF) [file pntd.0010893.s004.pdf]

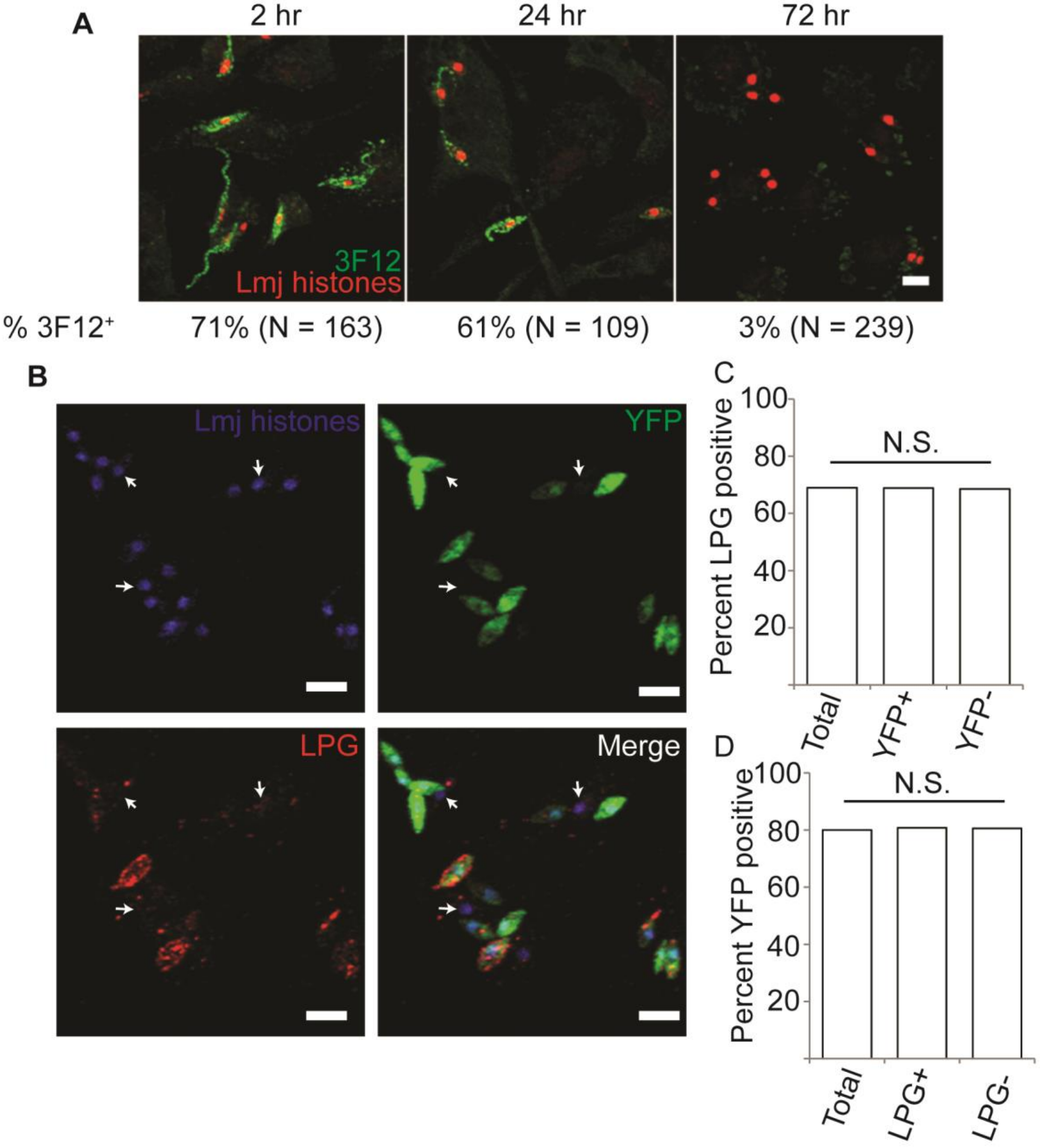

S4 Fig. LPG down-regulation is coincident with loss of metacyclic-specific arabinose-capped LPG but independent of YFP induction.

(A) PEMs were infected with *L. major* and harvested 2, 24, and 72 hours after infection. Ara-capped LPG is detected with mAB 3F12 (green), and parasite histones are shown in red. Data indicate the percent of parasites positive for 3F12 labeling out of the total number of parasites analyzed (N). Scale bar, 5 µm. **(B)** Representative image of parasites within BMM fixed 72 h post infection showing LPG and/or YFP positivity. Parasite nuclei are shown in blue. Arrows indicate LPG and YFP double-negative parasites. Scale bar, 5 µm. Analysis of images like that in (B) was performed to determine the LPG-positivity **(C)** or YFP-positivity **(D)** of total parasites or within the indicated parasite sup-populations. N = 362 parasites. N.S., not specific (Chi-square).
