# Supplementary material for: Quantitative single-cell analysis of Leishmania major amastigote differentiation demonstrates variably extended expression of the lipophosphoglycan (LPG) virulence factor in different host cell types
Source: PLoS Negl Trop Dis. 2022 Oct 27;16(10):e0010893. doi: 10.1371/journal.pntd.0010893 (PMC9642900; doi:10.1371/journal.pntd.0010893)
Supplement: S5 Fig — (A, B) L. major-infected BMM were cultured in the presence of BrdU for 72 hours prior to fixation, immunolabeling, and confocal microscopy. A representative image is shown in (A). Arrows indicate LPG-retaining parasites, which tend to be BrdU-negative as quantitated by Chi-square analysis in (B). Data shown include the percent of total parasites that are BrdU+, as well as the BrdU-positivity of parasites that are either LPG-negative or LPG-positive. Data, means ± S.E., n = 3 experiments, ***, P < 0.0001 (Chi-square; N = 848 parasites). (C, D) PEMs were infected with L. major metacyclic stage parasites for 24 h in the presence of EdU prior to fixation and staining to detect EdU (green), LPG (red) and parasite nuclei (blue). A Representative confocal image of a parasite showing double-positive labeling for EdU and LPG along with several EdU-negative parasites is shown in (C) with quantitation of EdU positivity amongst total parasites, LPG-positive, and LPG-negative parasites shown in (D). N.S., not significant per Chi-square analysis as in Fig 6. N = 429 parasites. (PDF) [file pntd.0010893.s005.pdf]

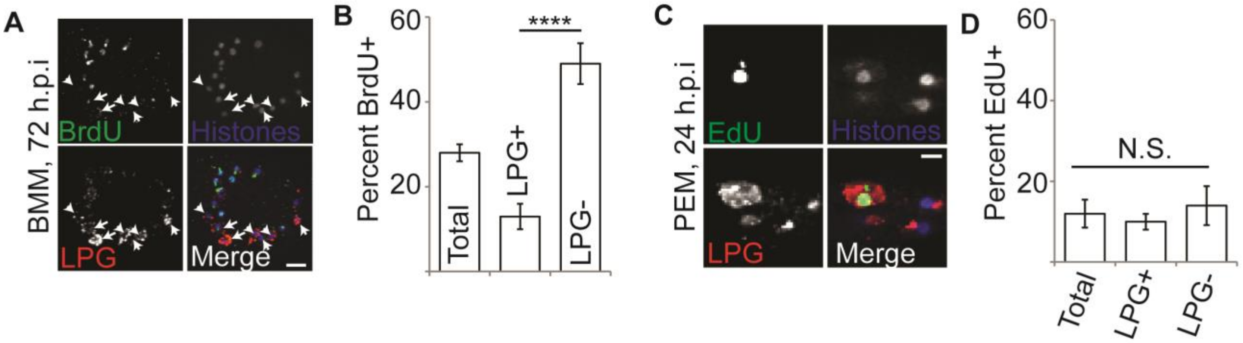

Figure S5. LPG loss is not prerequisite for DNA synthesis.

(A, B) *L. major*-infected BMM were cultured in the presence of BrdU for 72 hours prior to fixation, immunolabeling, and confocal microscopy. A representative image is shown in (A). Arrows indicate LPG-retaining parasites, which tend to be BrdU-negative as quantitated by Chi-square analysis in (B). Data shown include the percent of total parasites that are $BrdU^{+}$, as well as the BrdU-positivity of parasites that are either LPG-negative or LPG-positive. Data, means ± S.E., n = 3 experiments, ***, $P < 0.0001$ (Chi-square; N = 848 parasites). **(C, D)** PEMs were infected with *L. major* metacyclic stage parasites for 24 h in the presence of EdU prior to fixation and staining to detect EdU (green), LPG (red) and parasite nuclei (blue). A Representative confocal image of a parasite showing double-positive labeling for EdU and LPG along with several EdU-negative parasites is shown in (C) with quantitation of EdU positivity amongst total parasites, LPG-positive, and LPG-negative parasites shown in (D). N.S., not significant per Chi-square analysis as in Fig. 6. N = 429 parasites.
